# Angiotensin-converting enzyme 2 prevents lipopolysaccharide-induced rat acute lung injury *via* suppressing the ERK1/2 and NF-κB signaling pathways

**DOI:** 10.1038/srep27911

**Published:** 2016-06-15

**Authors:** Yingchuan Li, Zhen Zeng, Yongmei Cao, Yujing Liu, Feng Ping, Mengfan Liang, Ying Xue, Caihua Xi, Ming Zhou, Wei Jiang

**Affiliations:** 1 Department of Anesthesiology, Shanghai Jiaotong University Affiliated Sixth People’s Hospital, Shanghai 200233, China

## Abstract

Acute respiratory distress syndrome (ARDS) caused by severe sepsis remains a major challenge in intensive care medicine. ACE2 has been shown to protect against lung injury. However, the mechanisms of its protective effects on ARDS are largely unknown. Here, we report that ACE2 prevents LPS-induced ARDS by inhibiting MAPKs and NF-κB signaling pathway. Lentiviral packaged *Ace2* cDNA or *Ace2* shRNA was intratracheally administrated into the lungs of male SD rats. Two weeks after gene transfer, animals received LPS (7.5 mg/Kg) injection alone or in combination with Mas receptor antagonist A779 (10 μg/Kg) or ACE2 inhibitor MLN-4760 (1 mg/Kg) pretreatment. LPS-induced lung injury and inflammatory response were significantly prevented by ACE2 overexpression and deteriorated by *Ace2* shRNA. A779 or MLN-4760 pretreatment abolished the protective effects of ACE2. Moreover, overexpression of ACE2 significantly reduced the Ang II/Ang-(1-7) ratio in BALF and up-regulated Mas mRNA expression in lung, which was reversed by A779. Importantly, the blockade of ACE2 on LPS-induced phosphorylation of ERK1/2, p38 and p50/p65 was also abolished by A779. Whereas, only the ERK1/2 inhibitor significantly attenuated lung injury in ACE2 overexpressing rats pretreated with A779. Our observation suggests that AEC2 attenuates LPS-induced ARDS *via* the Ang-(1-7)/Mas pathway by inhibiting ERK/NF-κB activation.

Acute respiratory distress syndrome (ARDS) remains the major cause of mortality and morbidity in intensive care^1^,^2^. ARDS is a type of acute diffuse and inflammatory lung injury, which is caused by the release of pro-inflammatory cytokines and the recruitment of granulocytes and monocytes into the lung, leading to increased pulmonary vascular permeability and loss of aerated alveolus^3^,^4^. At present, the only effective therapy for ARDS is protective mechanical ventilation with low tidal volume of 6 mL/kg^5^. No specific and effective pharmacological intervention for ARDS is currently available^6^. Therefore, it is urgent to identify, validate, and develop pharmaceutical drugs for the treatment of ARDS.

Angiotensin-converting enzyme 2 (ACE2), a homologue of ACE, shares approximately 61% homology sequence with the catalytic domains of ACE but acts as an endogenous counter-regulator of ACE^7^. In contrast to ACE, which cleaves angiotensin (Ang) I into Ang II, ACE2 primarily hydrolyzes Ang II into Ang-(1-7). Ang II triggers vasoconstriction, inflammation, proliferation and apoptosis *via* binding to its specific Ang II type 1 receptor (AT1R), while Ang-(1-7) counteracts the effects of Ang II *via* its G protein-coupled receptor Mas^8^,^9^,^10^. Therefore, the balance of ACE and ACE2 influences the endogenous ratio of Ang II to Ang-(1-7), and consequently contributes to the regulation of the tension of vascular, as well as inflammatory response and organ function after injury^11^,^12^. Accumulating evidence indicate that ACE2 plays an important role in the pathophysiology of ARDS. ACE2 has been identified as a key receptor of coronavirus that causes the severe acute respiratory syndrome, and its level in human airway epithelia positively correlates with coronavirus infection^13^. Compared with wild type mice, *Ace2* knockout mice exhibited impaired exercise capacity, worse lung function and exacerbated lung fibrosis in model of bleomycin-induced lung injury^14^. Furthermore, in three animal models of ARDS, *Ace2* knockout mice showed severe lung disease, including enhanced vascular permeability and increased lung edema as compared to wild type mice^15^. Treatment with recombinant ACE2 effectively improved symptoms and attenuated arterial hypoxemia in a piglet model of lipopolysaccharide (LPS)-induced ARDS^16^. Thus, ACE2 plays a protective role in ARDS and is potential for the development as a drug for ARDS therapy; however, the underlying molecular mechanism by which ACE2 prevents ARDS remains elusive.

Previous studies have showed that the activation of mitogen-activated protein kinases (MAPKs) pathway is associated with the process of ARDS. The phosphorylation levels of p38 MAPK, extracellular signal-regulated kinase (ERK) and Jun N-terminal kinase (JNK) are all significantly increased in LPS-induced lung injury^17^. Consequently, inhibition of p38 MAPK or ERK efficiently attenuates LPS-induced pulmonary inflammatory response^18^,^19^. The activity of p38 MAPK in the lung has been associated with peritoneal sepsis-induced pulmonary edema and leukocyte infiltration^20^. Interestingly, Ferreira *et al*. recently found that activated cardiac ACE2 prevents hypertension-induced cardiac fibrosis by inhibiting ERK phosphorylation^21^. Moreover, AT1R blocker, telmisartan, exerts protective effects on heart failure through upregulating myocardial ACE2 level and inhibiting p38 MAPK, ERK and JNK phosphorylation^22^. The activation of MAPKs pathway is also involved in the regulation of *Ace2* mRNA expression in rat vascular smooth muscle cells^23^. In addition, LPS stimulation activates the NF-κB signaling pathway *via* binding to toll-like receptor 4 (TLR4), which is closely related to LPS-induced lung injury and inflammation^24^. Our previous *in vitro* study demonstrated that ACE2 prevented rat pulmonary microvascular endothelial cells (PMVECs) from LPS-induced apoptosis and inflammation through inhibiting the activation of JNK and NF-κB pathways^25^. Therefore, we hypothesize that ACE2 may protect against LPS-induced acute lung injury by inhibiting the MAPKs/ NF-κB pathway.

A variety of animal species have been used to study LPS-induced lung injury; however, there are intra-species differences in the biological response to LPS challenge, leading to inconsistency in published results. While rat and mouse models are the most widely used models for ARDS research, recent research has shown disparities of the LPS structures that are recognized by TLR4 between humans and mice, which may contribute to variability in the response to LPS-induced ARDS. Additionally, the relatively small mass of mice may prohibit the measurement of physiological parameters and also make it more difficult to obtain sufficient quantities of samples, such as blood, plasma and BALF^26^,^27^. In the present study, we applied a lentiviral-mediated gene delivery approach to overexpress or knock down ACE2 in rat lung tissue, and investigated whether pulmonary overexpression of ACE2 exerts beneficial effects against LPS-induced lung injury *via* suppressing the MAPKs/ NF-κB pathways.

## Materials and Methods

### Reagents

LPS (*Escherichia coli*, O127:B8) was purchased from Sigma-Aldrich (St. Louis, MO, USA). Mas receptor antagonist A779 was obtained from AbBiotech (San Diego, CA, USA). ACE2 inhibitor MLN-4760 was a product from EMD Millipore (Darmstadt, Germany). SB203580 (a specific inhibitor of p38 MAPK), PD98059 (a specific inhibitor of ERK1/2) and SP600125 (a specific inhibitor of JNK) were purchased from Santa Cruz Biotechnology (Delaware, CA, USA). Rabbit anti-ACE2, anti-p50, anti-p65, and mouse anti-phospho-p50, anti-phospho-p65 and anti-IκBα antibodies were procured from Santa Cruz Biotechnology. Rabbit anti-p38 MAPK, anti-phospho-p38 MAPK, anti-ERK1/2, anti-phospho-ERK1/2, anti-/JNK, anti-phospho-JNK, horseradish peroxidase (HRP)-conjugated goat anti-rabbit IgG, and horse anti-mouse IgG antibodies were purchased from Cell Signaling Technology (Danvers, MA, USA). TNF-α and IL-1β kits were purchased from Invitrogen (Eugene, OR, USA). AngII and Ang-(1-7) enzyme-linked immunosorbent assay (ELISA) kits were from Kamiya Biomedical (Seattle, WA, USA).

### Animals

Male Sprague-Dawley rats weighing 150–180 g were obtained from the Department of Laboratory Animal Science of Shanghai Jiaotong University (Shanghai, China). All animals were housed with free access to food and water in temperature-controlled rooms (25 ± 1 °C). All experimental procedures were approved by the Ethics Committee of Animal Research at the College of Medicine, Shanghai Jiaotong University (Shanghai, China), and were conducted in accordance with the international guidelines for care and use of laboratory animals.

### Generation of recombinant *Ace2* and small hairpin RNA (shRNA)-*Ace2* lentiviruses

According to the sequence of rat *Ace2* mRNA (NM_001012006.1), we designed a siRNA sequence (5′-GGTCACAATGGACAACTTC-3′) targeting the ACE2 coding region. The corresponding oligonucleotide templates of the shRNA were chemically synthesized and cloned into the pSIH1-H1-copGFP shRNA Vector (System Biosciences, California, USA), which was digested by BamHI and EcoRI and purified by agarose gel electrophoresis. A scrambled RNAi sequence (5′-GAAGCCAGATCCAGCTTCC-3′) was used as the negative control. The resultant plasmids were selected and confirmed by direct DNA sequencing.

Total RNA was extracted from rat PMVECs cells, and reversely transcribed into cDNA using M-MLV reverse Transcriptase (Takara BIO, Japan), which was used to amplify the ACE2 coding sequence using the following primers: Forward primer: 5′-GCTCTAGAGCCACCATGTCAAGCTCCTGCTGGC-3′ and Reverse primer: 5′- CGGGATCCTTAGAATGAAGTTTGAGC-3′. The product was purified and then ligated to the linear lentivector pcDNA-CMV-copGFP cDNA Vector (System Biosciences, USA). The ligation mixture was transformed into a competent DH5α strain and the positive clones were selected. The plasmids were extracted and then analyzed by PCR and sequencing.

According to the manufacturer’s instructions, the *Ace2* expression vector or shRNA vector and Lentivirus Package plasmid mix were co-transfected into 293 T producer cells using Lipofectamine™ 2000 (Invitrogen, CA, USA). The supernatants were collected 48 hours later and cleared by centrifugation and filtering through 0.45 μm PVDF membranes. Viral titer was evaluated by gradient dilution. The packaged lentiviruses were designated as Lv-ACE2 and Lv-ACE2-RNAi.

### Lentiviral transduction

For delivery of the Lenti-*Ace2* (recombinant lentivirus carrying *Ace2* cDNA) and Lenti-*Ace2*-RNAi (recombinant lentivirus carrying *Ace2* shRNA) into the lungs, rats underwent a midline incision to expose the trachea under isoflurane anesthesia. Briefly, anesthetized rats were placed in a supine position on an inclined platform (approximately 45°) and then the trachea was exposed surgically on the ventral side of neck. A 26# needle was inserted through the tracheal wall into the lumen just blow the larynx. Empty virus (control), the Lenti-*Ace2*, or Lenti-*Ace2*-RNAi viral particles (1 × 10^8^ if u/μl in 100 μl of phosphate-buffered saline) were directly injected into the trachea followed by 300 μl of air to enhance the spread of virus in the rat lungs. The same injection was performed 7 days later. One week after the last lentiviral treatment, animals were subjected to LPS administration.

### LPS-induced acute lung injury

Acute lung injury (ALI) was induced by single intravenous injection of LPS (7.5 mg/Kg) as previously described^28^. Control rats received 0.9% NaCl solution (500 μl) through the tail vein. In treatment groups, A779 (10 μg/Kg) or MLN-4760 (1 mg/Kg) was injected *via* rat tail vein 30 min before the induction of ALI. All animals were breathing spontaneously during the experimental protocol.

Eight hours after LPS administration, animals were anesthetized by intraperitoneal injection of pentobarbital sodium (50 mg/Kg) and euthanized by exsanguination. Serum sample were collected and stored at −80 °C. Broncho-alveolar lavage fluid (BALF) was collected from three lavage samples from the left lung with aliquots of 3 ml normal saline. Greater than 90% recovery of the saline was achieved. The retrieved BALF was pooled and centrifuged (300 g at 4 °C for 10 min). The supernatant was stored in aliquots at −80 °C. The right lung was fixed immediately in 4% paraformaldehyde.

To further investigate the role of MAPKs pathways in development of ARDS and the effects of ACE2 overexpression, MAPKs specific inhibitors (SB203580, PD98059 and SP600125, 10 mg/Kg) were pretreated intraperitoneally at the time 10 min before LPS administration.

### Histopathology

The embedded lung tissues were cut into 4 μm-thick sections and stained with hematoxylin-eosin for microscopic observation. The degree of lung injury was semi-quantitatively scored as described by Murakami and colleagues^29^. Briefly, the lung injury score, including edema, inflammation, and hemorrhage, which was graded on the following scale: normal (0), light (1), moderate (2), strong (3), and severe (4). Analysis was conducted by a pathologist blinded to the experimental group. The values of each of the three parameters analyzed were added. The final lung injury score was the average score calculated within each experimental group.

### Measurement of protein concentration and inflammatory mediators in BALF

For assessment of lung permeability in rats, the protein concentration in the BAL fluid was measured. Briefly, BALF samples (100 μl) from the left lung were centrifuged at 4 °C, 1500 g for 5 min, and protein concentration in the supernatant was quantified by BCA protein assay (Pierce, IL, USA).

BALF levels of IL-1β, TNF-α, AngII and Ang-(1-7) were measured using ELISA assay in accordance with the manufacturer’s instructions.

### Western blot analysis

Total protein was extracted from the frozen lung tissue using T-PER Tissue Protein Extraction Reagent (Pierce, IL, USA). The equal amounts of protein (100 μg) were run on a 10% SDS-PAGE gel and transferred onto polyvinylidene difluoride membranes (IPVH00010, Millipore). The membranes were blocked with 5% skim milk in TBST at room temperature for 2 h and then incubated with primary antibody against rat ACE2 (1:800), ERK1/2 (1:600), phosphorylated ERK1/2 (1:400), JNK (1:500), phosphorylated JNK (1:500), p38 MAPK (1:800), phosphorylated P38 MAPK (1:600), p65/p50 (1:500), phosphorylated p65/p50 (1:300), IκBα (1:500) and β actin (1:1000) at 4 °C overnight. After 3 washes with TBST, the blots were incubated in secondary HRP-conjugated anti-mouse/rabbit IgG at room temperature for 1 h. After washing with TBST, the membranes were developed with an ECL detection kit (Pierce, IL, USA) and imaged with X-ray films.

### Real-time PCR

Total RNA was extracted from lung tissues with Trizol reagent (15596-018, Invitrogen, OR, USA). Quantitative real-time PCR was performed using the Thermal Cycler Dice Real Time System (TP800, Takara, Japan). Briefly, a solution of 2 μg RNA mixed with 2 μl of 50 μM Oligo (dT). The primers were diluted to a final volume of 10 μl with RNase free distilled water (dH_2_O), incubated at 70 °C for 10 min, and then kept on ice for 2 min. The solution was mixed with 4 μl of 5× buffer, 1 μl of 10 mM dNTP mixture (D4030RA, Takara), 1 μl of 40 U/μl Ribonuclease inhibitor (D2310A, Takara), 1 μl of 200 U/μl RNase M-MLV, and diluted to 20 μl with RNase dH_2_O, then incubated at 42 °C for 60 min, and 70 °C for 15 min. Next, the reaction mixture, containing 2 μl cDNA, 0.4 μl primer F, 0.4 μl primer R, and 10 μl SYBR premix Ex Taq (DRR041A, Takara) was diluted to 20 μl with RNase dH_2_O and kept at 95 °C for 5 min. The reaction conditions were as follows: 40 cycles of 95 °C for 10 sec, 60 °C for 20 sec and 72 °C for 20 sec. The 2^−∆∆Ct^ method was used to analyze the relative mRNA level of target gene as the fold change normalized to that of beta-actin gene and relative to the sham group. The following primers were used: *Ace2*, Forward primer: 5′-GCTCCTGCTGGCTCCTTCTCA-3′, Reverse primer: 5′-GCCGCAGCCTCGTTCATCTT-3′, *Mas*, forward primer: 5′-CATTCGTCTGTGCCCTCCTGTG-3′, reverse primer: 5′-GGCCCATCTGTTCTTCCGTATCTT-3′; *β-actin*, forward primer: 5′-CCTAAGGCCAACCGTGAAAAGATG-3′, reverse primer: 5′-GTCCCGGCCAGCCAGGTCCAG-3′.

### Statistical analyses

Statistical analyses were performed with the Prism software package (GraphPad v5, San Diego, CA, USA). All values are presented as mean ± standard deviation (SD). Data were analyzed using one-way ANOVA and the Newman-Keuls test for multiple comparisons. A *P*-value less than 0.05 was considered statistically significant.

## Results

### Lentiviral-mediated gene transfer efficiently increases or down-regulates *Ace2* mRNA and ACE2 protein expression in rat lung tissue

**Figure 1:**
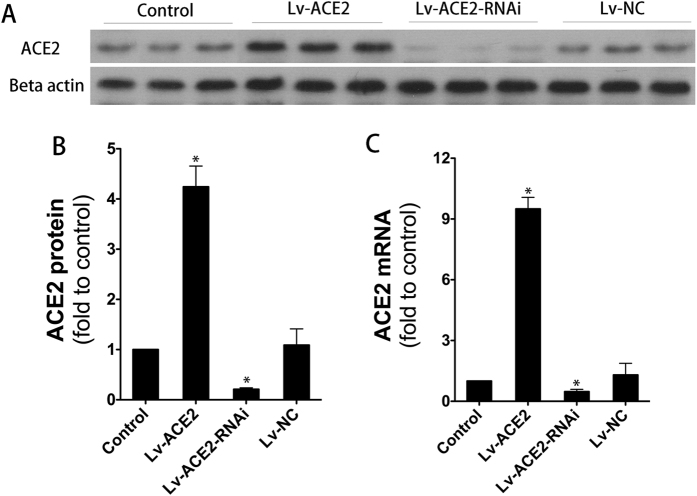
Efficiency of gene transfer at two weeks after Lenti-Ace2 and Lenti-Ace2-RNAi delivery. (**A,B**) Western-blotting analysis showed that ACE2 expression of rat lung tissue was significantly increased in the Lv-ACE2 group and decreased in the Lv-ACE2-RNAi group, as compared with the control group. (**C**) Quantitative analysis of ACE2 mRNA levels by using RT-PCR. Lung ACE2 mRNA levels were significantly increased by Lenti-ACE2 transfection, which were suppressed by Lenti-ACE2-RNAi transduction. The Data are represented as mean ± SD. *p < 0.05, versus control group (n = 3, per group).

Compared with control animals, ACE2 mRNA and protein levels in the lung tissue of the Lv-ACE2 group (rat lung transfected with Lenti-*Ace2*) were increased by 9.47 and 4.21 fold, respectively; in contrast, the ACE2 mRNA and protein levels in Lenti-*Ace2*-RNAi infected rat lungs (Lv-ACE2-RNAi group) were decreased by 52% and 80%. There were no significant differences in ACE2 mRNA and protein expression between the control group and Lv-NC group (rat lung transfected with recombinant lentivirus carrying negative control shRNA). These results showed high efficiency of *Ace2* or *Ace2*-shRNA gene transfer into rat lung *via* intratracheal injection. (Fig. 1A–C)

### ACE2 prevents rat from LPS-induced acute lung injury

**Figure 2:**
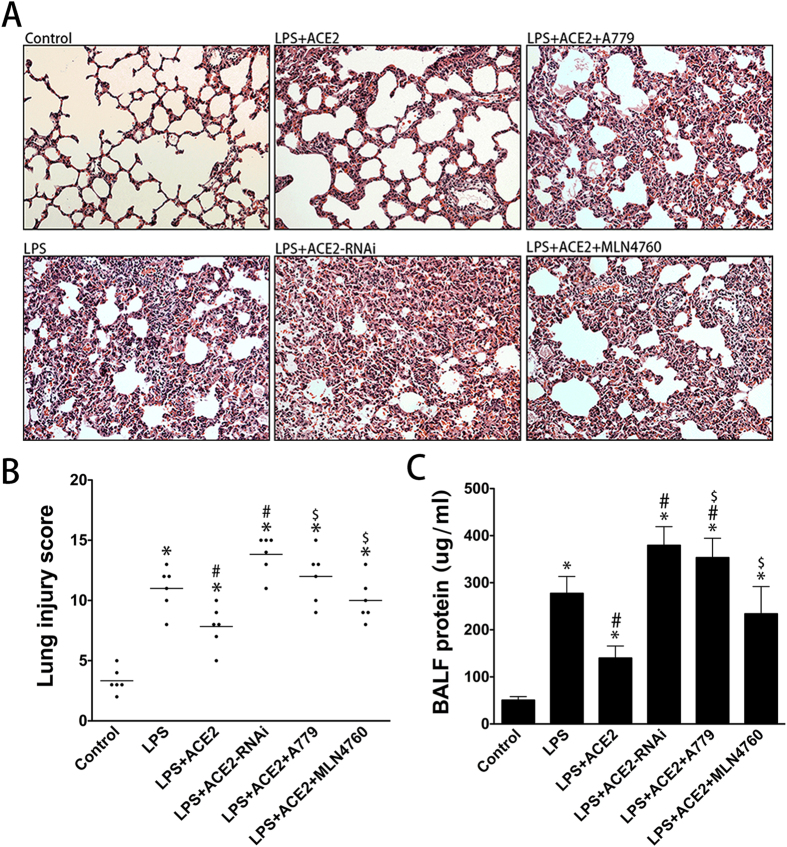
Effects of ACE2 and different treatments on LPS-induced lung injury. (**A**) Representative photographs of the lung tissues stained with hematoxylin and eosin (original magnification x20). (**B**) Morphological changes in lung section semi-qualified using lung injury score. (**C**) Protein concentrations in BALF in various treatment groups as determined by ELISA. Data are represented as mean ± SD. *p < 0.05, versus control group; ^#^p < 0.05, versus LPS group; ^$^p < 0.05, versus ACE2 group (n = 6, per group).

After 8 hours, LPS treatment resulted in acute lung injury as demonstrated by edema, inflammation, and hemorrhage (Fig. 2A). The lung injury score (Fig. 2B) and BALF protein level (Fig. 2C) of the LPS group were significantly increased as compared with the control group. ACE2 overexpression in rat lung markedly attenuated LPS-induced lung injury and decreased the lung injury score and BALF protein level. In sharp contrast, silencing ACE2 noticeably deteriorated LPS-induced lung injury as evidenced by increased lung injury score and BALF protein level. Furthermore, pretreatment with either A779, a potent and selective antagonist of Ang-(1-7) *via* competitively binding with its specific Mas receptor, or MLN-4760, the ACE2 inhibitor, significantly inhibited the protective effects of ACE2 on LPS-induced lung injury. The lung injury score and BALF protein level of the LPS+ACE2+A779 group were significantly higher than that of LPS+ACE2 group, suggesting that ACE2 protects rat from LPS-induced acute lung injury, partially *via* Mas-mediated signaling.

### ACE2 attenuates LPS-induced inflammatory response and reverses Ang II/Ang-(1-7) ratio in BALF

**Figure 3:**
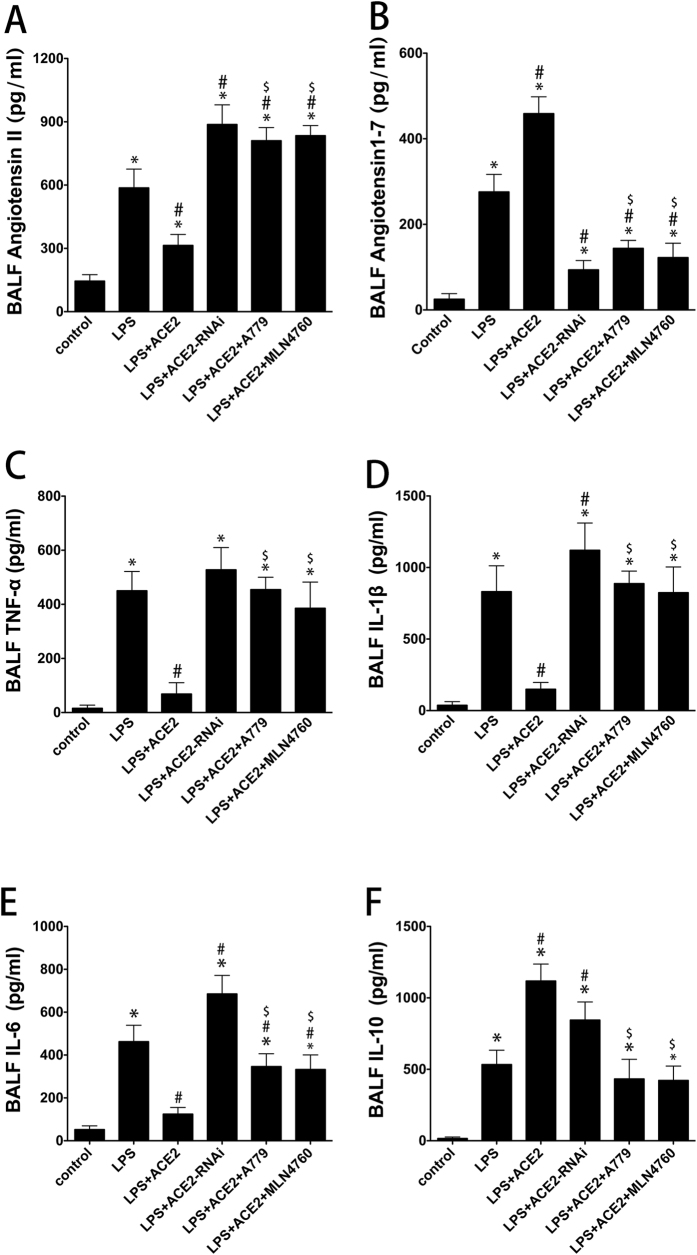
Effects of ACE2 and different treatments on LPS-induced secretion of inflammatory mediators in BALF. (**A**) LPS administration caused a significant increase of Ang II, which was markedly attenuated by ACE2 overexpression and aggregated by ACE2 knockdown. (**B**) LPS exposure also caused a significant increase of Ang-(1-7), which was further up-regulated by ACE2 overexpression and reduced by ACE2 knockdown. Pretreatment with A779 or MLN-4760 both reversed the changes of Ang II and Ang-(1-7) that resulted from ACE2 overexpression. (**C–F**) The levels of TNF-α, IL-1β, IL-6 and IL-10 were significantly increased after 8 hours of LPS injection. ACE2 overexpression markedly suppressed the TNF-α, IL-1β and IL-6 secretion and increased IL-10 level, which were noticeably abolished by pretreatment with A779 or MLN-4760. The increased levels of TNF-α, IL-1β IL-6 and IL-10 caused by LPS administration were all further promoted by ACE2 knockdown. Data are represented as mean ± SD. *p < 0.05, versus control group; ^#^p < 0.05, versus LPS group; ^$^p < 0.05, versus ACE2 group (n = 6, per group).

After LPS administration, the levels of both Ang II (Fig. 3A) and Ang-(1-7) (Fig. 3B) in BALF significantly increased as compared with the control group. ACE2 overexpression significantly reduced the BALF Ang II concentration and enhanced Ang-(1-7) level. The ratio of Ang II to Ang-(1-7) markedly dropped from 2.12 in the LPS group to 0.68 in the LPS+ACE2 group. A779 or MLN-4760 pretreatment in Lv-*Ace2* transduced rat both reversed the decrease of Ang II level, and increasing Ang-(1-7) level in BALF. The ratio of Ang II to Ang-(1-7) notably increased to 5.63 and 6.83 in LPS+ACE2+A779 group and LPS+ACE2+MLN4760 group, respectively. In addition, ACE2 RNAi also significantly increased Ang II level and decreased Ang-(1-7) level, with the ratio of Ang II to Ang-(1-7) increased to 9.48, which was the highest level in all groups.

The TNF-α (Fig. 3C), IL-1β (Fig. 3D) and IL-6 (Fig. 3E) concentrations in the BALF of the rats exposed to LPS were significantly higher than that in control group. LPS-induced secretion of TNF-α, IL-1β and IL-6 in BALF were dramatically inhibited by ACE2 overexpression, but further promoted by ACE2 knockdown in rat BALF. Treatment with A779 or MLN-4760 prior to LPS injection resulted in an obvious increase of TNF-α, IL-1β and IL-6 in BALF of rat transduced with Lenti-*Ace2*. The concentration of TNF-α and IL-1β in BALF of the LPS+ACE2+A779 group or the LPS+ACE2+MLN-4760 group were similar to that of the LPS group. These results demonstrated that LPS mainly promoted the generation of Ang II and enhanced the cytokines secretion, all of which were abolished by ACE2 overexpression.

ACE2 overexpression in rat lung significantly enhanced LPS-induced IL-10 secretion in BALF, which was abolished by A779 or MLN4760 pretreatment. Interestingly, ACE2 knockdown in rat lung also promoted IL-10 level in BALF after LPS administration. These results suggest that the anti-inflammatory response may be also triggered in lung by LPS exposure to counter the effects of pro-inflammatory cytokines, and ACE2 promotes anti-inflammatory response *via* Ang-(1-7)/Mas pathway.

### ACE2 overexpression reversed LPS-induced ACE2 decrease and enhanced Mas mRNA increase in lung

**Figure 4:**
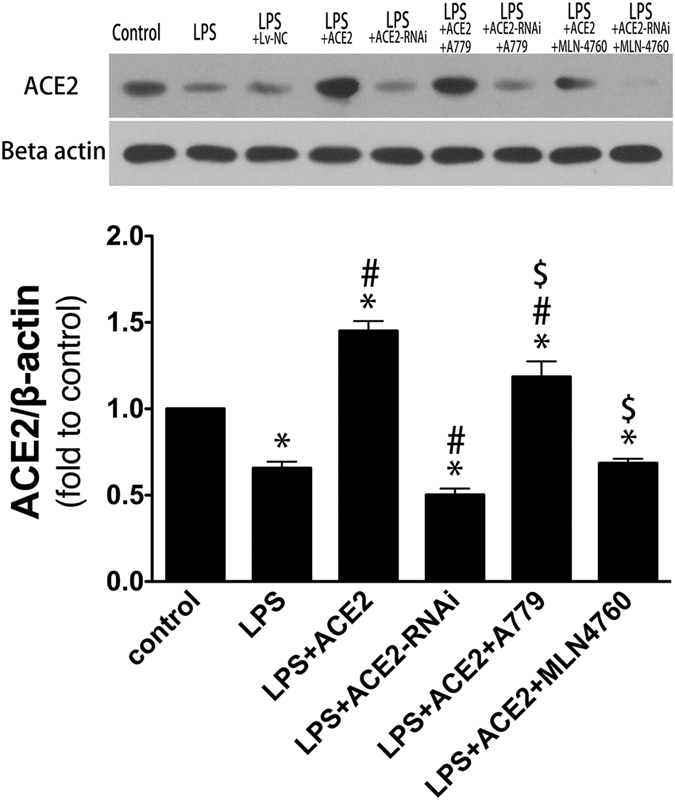
Effects of ACE2 and different treatments on the expression of ACE2 protein in lung tissue. LPS exposure caused a significant decrease of ACE2 protein expression in lung tissue, which was obviously up-regulated by Lenti-*Ace2* transfection and down-regulated by Lenti-*Ace2*-RNAi. Up-regulation of ACE2 protein in lung tissue transduced with Lenti-*Ace2* was decreased in rat treated with A779 prior to LPS exposure and completely suppressed by MLN-4760 treatment before LPS administration. Data are represented as mean ± SD. *p < 0.05, versus control group; ^#^p < 0.05, versus LPS group; ^$^p < 0.05, versus ACE2 group (n = 6, per group).

LPS administration resulted in a significant decrease of ACE2 protein in rat lungs, which was up-regulated by Lenti-*Ace2* transduction and attenuated by ACE2-RNAi (Fig. 4). The up-regulated ACE2 protein expression in rat lung by Lenti-*Ace2* was suppressed by A779 or MLN-4760 pretreatment before LPS injection. The ACE2 expression in LPS+ACE2+MLN-4760 group was similar to that in the LPS group.

**Figure 5:**
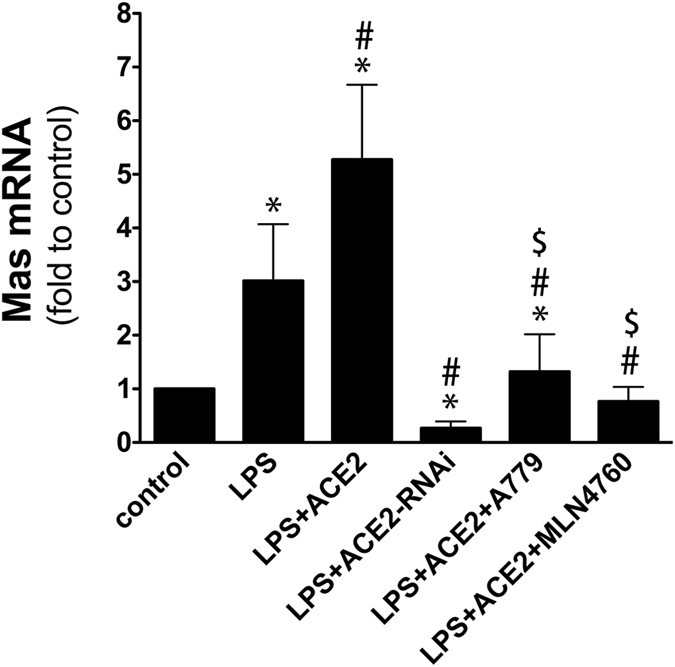
Effects of ACE2 and different treatments on the expression of Mas mRNA in lung tissue. LPS administration resulted in a significant increase of *Mas* mRNA expression in lung tissue, which was further up-regulated by Lenti-*Ace2* transfection and completely suppressed by Lenti-*Ace2*-RNAi. *Mas* mRNA expression in lung tissue of rat transduced with Lenti-*Ace2* was markedly reduced by A779 or MLN-4760 treatment before LPS injection. Data are represented as mean ± SD. *p < 0.05, versus control group; ^#^p < 0.05, versus LPS group; ^$^p < 0.05, versus ACE2 group (n = 6, per group).

*Mas* mRNA was up-regulated in lung tissue of rats that were exposed to LPS. ACE2 overexpression caused a significant further increase of *Mas* mRNA in LPS stimulated rat lung, while ACE2 knockdown markedly suppressed the *Mas* mRNA expression. Treatment with A779 or MLN-4760 before LPS administration significantly reduced the expression of *Mas* mRNA in Lenti*-Ace2* transduced rat lung. There was no significant difference in *Mas* mRNA expression between LPS+ACE2+MLN-4760 group and control group (Fig. 5).

### ACE2 suppresses the MAPKs pathway that mediates LPS-induced lung injury

**Figure 6:**
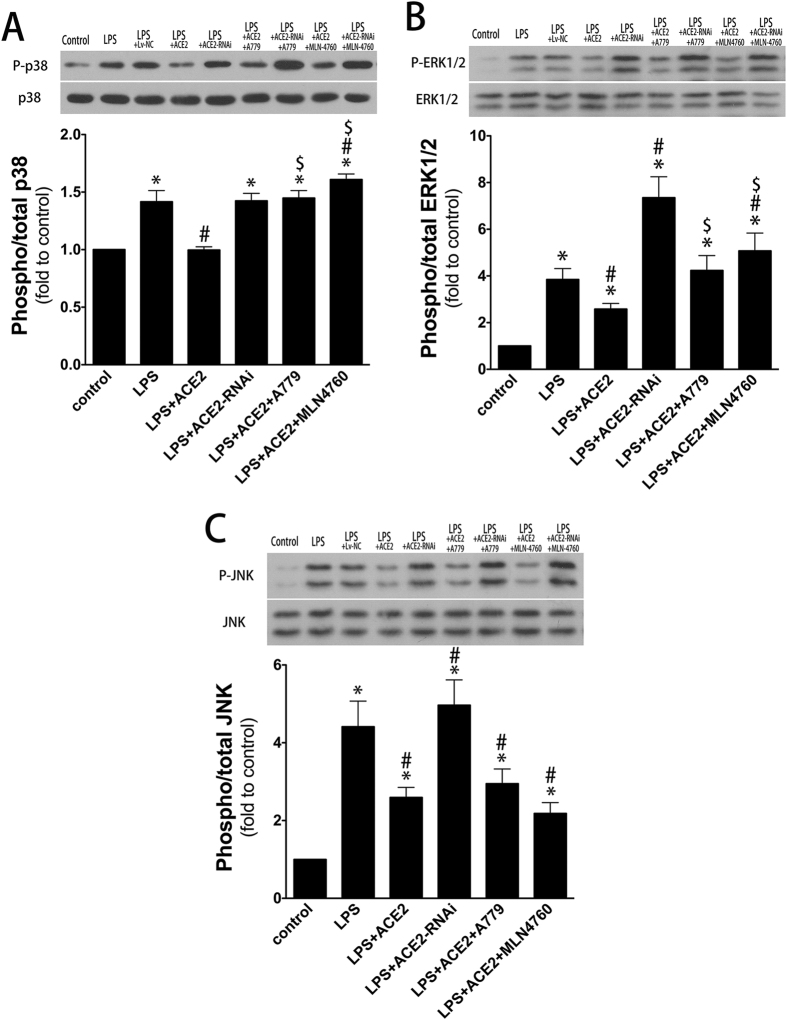
Effects of ACE2 and different treatments on the phosphorylation of MAPKs in lung tissue. LPS exposure caused a marked increase of the phosphorylation levels of p38 MAPK (**A**), ERK1/2 (**B**) and JNK (**C**), which was significantly suppressed by ACE2 overexpression in rat lung. Pretreatment with A779 or MLN-4760 completely abolished the inhibitory effects of ACE2 overexpression on LPS-induced p38 MAPK and ERK1/2 phosphorylation, but did not affect the level of JNK phosphorylation. ACE2 RNAi in rat lung significantly enhanced the LPS-induced ERK1/2 and JNK phosphorylation but did not change p38 MAPK phosphorylation. Data are represented as mean ± SD. *p < 0.05, versus control group; ^#^p < 0.05, versus LPS group; ^$^p < 0.05, versus ACE2 group (n = 6, per group).

The Western blot results showed that LPS administration induced the phosphorylation of p38 MAPK (Fig. 6A), ERK1/2 (Fig. 6B) and JNK (Fig. 6C). ACE2 overexpression significantly suppressed the phosphorylation levels of p38 MAPK, ERK1/2 and JNK in lung tissue of rats receiving LPS. The inhibitory effects of ACE2 overexpression on LPS-induced p38 MAPK and ERK1/2 phosphorylation were completely abolished by pretreatment with A779 or MLN-4760. Meanwhile, there was no significant difference in the JNK phosphorylation in LPS+ACE2+A779 or LPS+ACE2+MLN-4760 as compared to the LPS+ACE2 group. Additionally, ACE2 knock down in rat lung significantly enhanced the LPS-induced increase of ERK1/2 and JNK phosphorylation in rat lung and there was no significant difference in p38 MAPK phosphorylation level between the LPS+ACE2-RNAi group and the LPS group (Fig. 6).

### Inhibition of the MAPKs pathways reverses the blockade of A779 or MLN4760 on the protective effects of ACE2 overexpression

**Figure 7:**
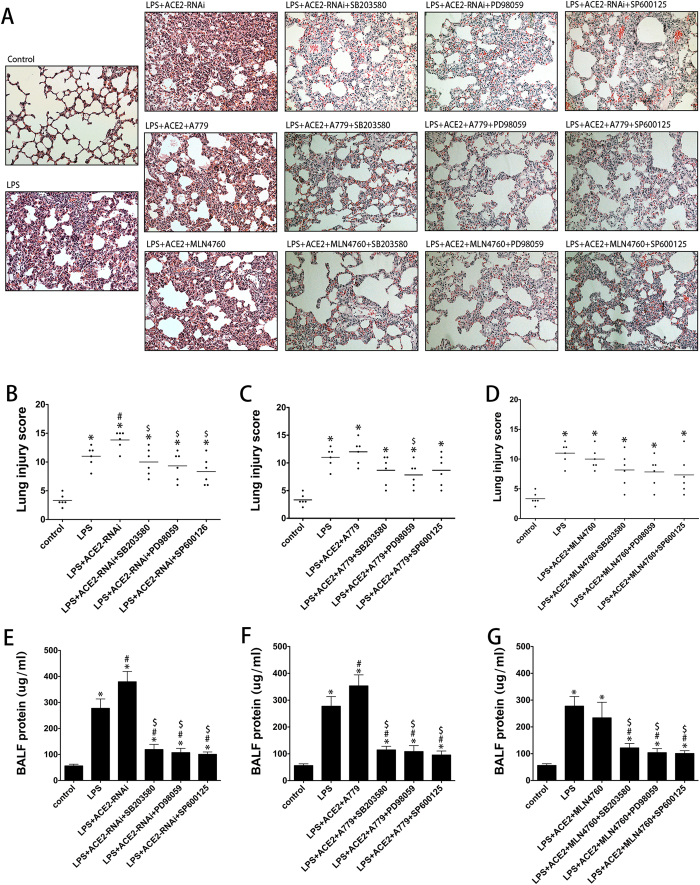
Effects of MAPKs inhibitors on LPS-induced lung injury. (**A**) Representative photographs of the lung tissues stained with hematoxylin and eosin (original magnification x20). (**B–D**) Morphological changes in lung section semi-qualified using lung injury score. (**E–G**) Protein concentrations in BALF in various treatment groups are determined by ELISA. Data are represented as mean ± SD. *p < 0.05, versus control group; ^#^p < 0.05, versus LPS group; ^$^p < 0.05, versus (**B,E**) LPS+ACE2-RNAi group, (**C,F**) LPS+ACE2+A779 group, (**D,G**) LPS+ACE2+MLN4760 group (n = 6, per group).

**Figure 8:**
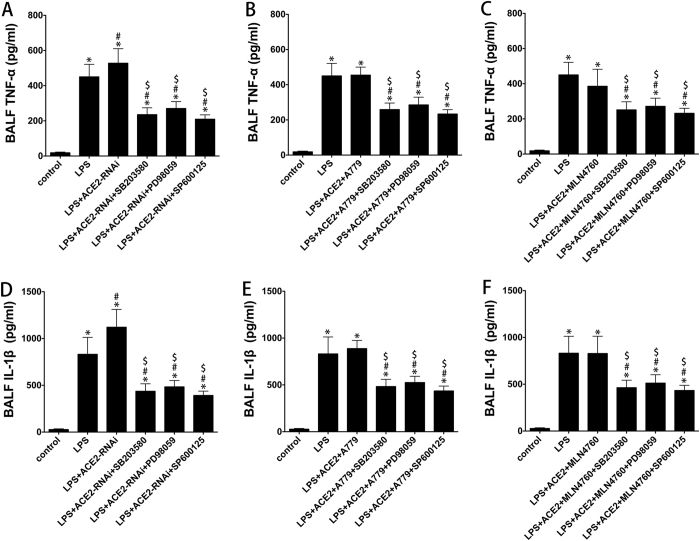
Effects of MAPKs inhibitors on LPS-induced lung cytokine secretion. In LPS+ACE2-RNAi group, SB203580, PD98059 or SP600125 pretreatment significantly suppressed LPS-induced TNF-α and IL-1β secretion in rat BLAF. In Lenti-*Ace2* infected rats, pretreatment with SB203580, PD98059 or SP600125 reversed the blockade effects of A779 or MLN4760 on ACE2 overexpression, and markedly attenuated LPS-induced cytokine secretion in BALF. Data are represented as mean ± SD. *p < 0.05, versus control group; ^#^p < 0.05, versus LPS group; ^$^p < 0.05, versus (**A**,**D**) LPS+ACE2-RNAi group, (**B**,**E**) LPS+ACE2+A779 group, (**C**,**F**) LPS+ACE2+MLN4760 group (n = 6, per group).

Pretreatment with specific MAPKs inhibitors in Lenti-*Ace2*-RNAi infected rats attenuated LPS-induced lung injury as evidenced by reduced lung injury score and BALF protein level (Fig. 7A,B and E). The BALF protein levels in rats of the LPS+ACE2+A779 group and LPS+ACE2+MLN4760 group were significantly reduced by SB203580, PD98059 or SP600125 pretreatment. The LPS-induced lung injury was also attenuated by MPAKs inhibitors prior to LPS administration in rats of these two groups (Fig. 7A,F and G). However, the evaluation of lung injury score showed that there was no statistical difference between the rats with or without MPAKs inhibitor pretreatment in the LPS+ACE2+A779 group and LPS+ACE2+MLN4760 group, except that the rats of LPS+ACE2+A779 group pretreated with PD98059 showed a significant decrease in lung injury score (Fig. 7C,D). In addition, the BALF cytokine TNF-α and IL-1β were dramatically reduced by specific MAPKs inhibitors pretreatment in the rats from the LPS+ACE2-RNAi, LPS+ACE2+A779 and LPS+ACE2+MLN4760 groups (Fig. 8). These results indicate that blocking the MAPKs pathway can remarkably attenuate LPS-induced inflammatory response in Lenti-*Ace2*-RNAi infected rats and A779 or MLN-4760 pretreated rats with ACE2 overexpression. Furthermore, the histological evaluation of whole lung injury demonstrated that pretreatment with the specific MAPKs inhibitors significantly attenuated LPS-induced lung injury in Lenti-*Ace2*-RNAi infected rats, but did not significantly reverse the blockade of MLN4760 on the effects of ACE2 overexpression in preventing LPS-induced lung injury. Specially, inhibiting the ERK1/2 pathway significantly attenuated both LPS-induced cytokine secretion and lung injury in ACE2 overexpression rats pretreated with Mas receptor antagonist.

### ACE2 inhibits LPS-induced activation of NF-κB pathway in rat lung

**Figure 9:**
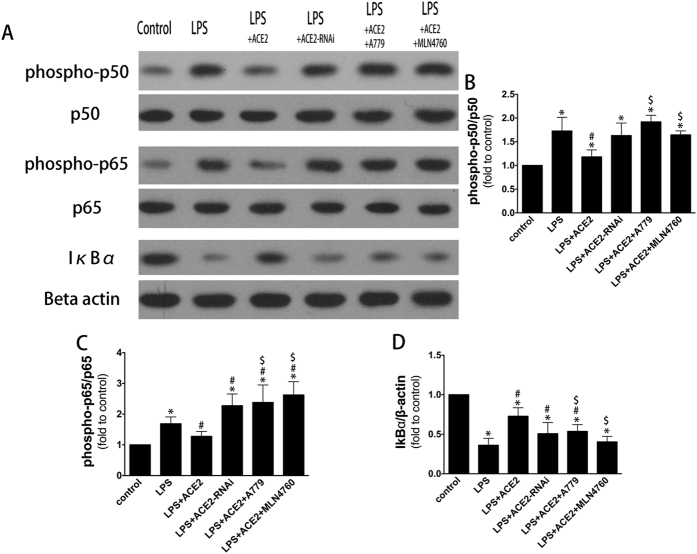
Effects of ACE2 and different treatments on LPS-induced activation of NF-κB pathway. LPS exposure caused a marked increase in the phosphorylation of NF-κB p50 and p65 and decrease of IκBα expression, which was significantly reversed by ACE2 overexpression in the rat lung. Pretreatment with A779 or MLN-4760 completely abolished the inhibitory effects of ACE2 overexpression on LPS-induced NF-κB p50 and p65 phosphorylation and reduced IκBα expression. ACE2 RNAi in the rat lung significantly enhanced the LPS-induced NF-κB p65 phosphorylation but did not change NF-κB p50 phosphorylation. Data are represented as mean ± SD. *p < 0.05, versus control group; ^#^p < 0.05, versus LPS group; ^$^p < 0.05, versus ACE2 group (n = 6, per group).

Eight hours after LPS administration, the NF-κB p50 and p65 phosphorylation level significantly increased and the IκBα expression clearly decreased in lung as compared to control rats. ACE2 overexpression in the rat lung markedly suppressed LPS-induced NF-κB p50 and p65 phosphorylation and enhanced the expression of IκBα, which were completely abolished by A779 or MLN4760 pretreatment. Furthermore, the NF-κB p65 phosphorylation level was higher in the LPS+ACE2+A779 group and LPS+ACE2+MLN4760 group than the LPS group. As compared with the LPS group, ACE2 down-regulation further enhanced LPS-induced increase of NF-κB p65 phosphorylation level in lung tissue. There was no difference in NF-κB p50 phosphorylation level between the LPS group and LPS+ACE2-RNAi group. Meanwhile, the IκBα expression level in the LPS+ACE2-RNAi group was significantly lower than that in the control group, but still higher as compared with the LPS group (Fig. 9).

## Discussion

In this study, we demonstrated that LPS administration evoked severe lung injury, characterized by increased inflammatory cell infiltration, edema, and hemorrhage in the interstitium and alveolus, as well as increased TNF-α and IL-β levels in BALF. Our results also showed that LPS administration significantly reduced ACE2 expression in the rat lung, whereas overexpression ACE2 significantly attenuated LPS-induced lung injury and suppressed inflammatory response, which was abolished by ACE2 inhibitor. Further, ACE2 knockdown caused a marked deterioration of lung injury and increase of cytokine secretion in rats receiving LPS injection. Together, these findings suggest that ACE2 in local lung tissue prevents LPS-induced lung injury and inflammation, and may be useful as a therapeutic agent targeting ARDS.

Activation of renin-angiotensin system plays a central role in the pathophysiology of ARDS, and suppression of the ACE/Ang II/AT1R axis has been shown to improve the symptoms of ARDS^28^,^30^,^31^,^32^. In different ARDS models, loss of ACE2 in mice resulted in more aggravated lung injury, whereas recombinant ACE2 attenuated the symptoms of ARDS in Ace2-knockout mice, especially in wild type mice^15^. The lung injury in ARDS mouse model caused by limb ischemia-reperfusion (LIR) was deteriorated by *Ace2* depletion while protected by *Ace2* transgene, and the changes of ACE2 expression in lung tissue were accompanied by alteration of Ang II/Ang-(1-7) ratio^33^. Prior studies have shown that down-regulation of ACE2 expression in lung and increased serum Ang II level are associated with severity of respiratory syncytial virus H7N9 or H5N1-induced ARDS, which is also ameliorated by administration of recombinant human ACE2^34^,^35^,^36^. Moreover, the decrease of ACE2 activity in BALF of ventilated animals exposed to LPS was correlated with enhanced BALF Ang II but reduced Ang-(1-7) levels^11^. Similar to these previous studies, our results showed that ACE2 overexpression in rat lung reduced the BALF Ang II level and further increased Ang-(1-7) level in animals treated with LPS. The ratio of Ang II to Ang-(1-7) in BALF was significantly decreased by ACE2 overexpression while markedly increased by ACE2 knockdown. Despite the most of evidences suggest an decrease of Ang-(1-7) level in lung tissue during the development of ARDS, there are some conflicting results from published literatures. The Ang-(1-7) level in BALF increases during mechanical ventilation-induced rat lung injury but decreases after 24 hours of LPS administration^11^. In a mouse ARDS model, Ang-(1-7) in lung tissue was shown a decrease after 24 hours of LPS challenge^37^. In the early stage of LIR-induced ARDS, the lung tissue Ang-(1-7) level significantly increased at 1 hour and persisted up to 12 hours after reperfusion^33^. In the present study, we found that LPS administration caused a significant increase of Ang II and also an unexpected increase of Ang-(1-7) in BALF. In renin-angiotensin system, Ang-(1-7) is mainly generated by ACE2 hydrolyzing Ang II, but it also can be generated by ACE catalyzing Ang-(1-9). It is possible that the increased levels of Ang-(1-7) in lung tissue may play a compensatory role in the initial development of ARDS.

The discovery that Ang-(1-7), the major product of ACE2, stimulates downstream Mas receptors to oppose the effects of Ang II/AT1R^38^,^39^, suggesting that Ang-(1-7) may contribute to the protective effects of ACE2. In support of this hypothesis, Ang-(1-7) overexpression in lung tissue prevented bleomycin-induced lung injury, and blockade of the Mas receptor abolished the beneficial effects of Ang-(1-7) against monocrotaline-induced pulmonary hypertension^40^. Ang-(1-7) or Mas agonist, AVE0991, prevent ventilator- or acid aspiration-induced lung injury, which was reversed by a Mas receptor antagonist^41^. In the present study, we demonstrated that Mas receptor antagonist effectively abolished the protective effects of ACE2 overexpression in the lung of rats exposed to LPS. In addition, Mas receptor antagonist also reversed the decrease of Ang II and increase of Ang-(1-7) in BALF of Lenti-*Ace2* transduced rats, elevating the Ang II/Ang-(1-7) ratio. These results indicate that the protective effects of ACE2 on LPS-induced lung injury primarily depend on the Ang-(1-7)/Mas pathway.

Recent study has found that infusion of Ang-(1-7) enhances *Mas* mRNA expression in a bleomycin-induced lung injury model^42^. Furthermore, overexpression of ACE2 in rostral ventrolateral medulla of spontaneously hypertensive rats increased the expression of Mas^43^. In this study, we found that ACE2 overexpression resulted in a significant further increase of *Mas* mRNA expression in LPS-stimulated rat lungs, while ACE2 knockdown remarkably suppressed *Mas* mRNA expression. Moreover, blockade of Mas receptor not only reduced the expression of ACE2 in lung tissue but also significantly suppressed *Mas* mRNA expression. These data suggest that the protective effects of ACE2/Ang-(1-7) against lung injury not only depend on Mas signaling cascade but also up-regulating Mas receptor expression.

The underlying molecular mechanism of ACE2 protects against ARDS remains elusive. Previous studies indicated that activation of MAPKs signaling promoted the progression of ARDS^17^, but the functional linkage of the MAPKs pathways with ACE2/Ang-(1-7)/Mas signaling axis in LPS-induced lung injury has not been elucidated. Our current study showed that LPS administration stimulated the phosphorylation of P38 MAPK, ERK1/2 and JNK, which was significantly reduced by ACE2 overexpression in lung tissue. Moreover, *Ace2* shRNA enhanced the phosphorylation of ERK1/2 and JNK in rat lung. Either Mas receptor antagonist or ACE2 inhibitor completely abolished the inhibitory effects of AEC2 on LPS-induced p38 MAPK and ERK1/2 phosphorylation. Combined with the changes of Ang II/Ang-(1-7) and *Mas* mRNA, these results suggest that the protection of LPS-induced ARDS by ACE2 relies on the Ang-(1-7)/Mas pathway to inhibit the signaling cascade of p38 MAPK and ERK1/2. Similar to our findings, Xue T *et al*. found that overexpression of ACE2 in rat lung attenuated smoking-induced lung inflammation *via* inhibition of p38 MAPK signaling^44^. Song B *et al*. reported that recombinant human ACE2 prevented Ang II-mediated myocardial injury through blocking the activation of ERK1/2^45^. In addition, it has been shown that ACE2 regulates the balance of AngII/Ang(1-7) to prevent alveolar epithelial cells apoptosis and JNK phosphorylation, while pretreatment with A779 abolishes the anti-apoptotic effect of Ang-(1-7) and its inhibition of JNK phosphorylation^46^. ACE2 over-expression in PMVECs protects LPS-induced apoptosis and suppresses p38 MAPK/JNK phosphorylation, which was attenuated by pretreatment with A779^25^. These data indicate that the suppression of MAPKs activation by the ACE2/Ang-(1-7)/Mas signaling pathway is cell type dependent.

To investigate whether the MAPKs pathway plays a role in the protective effect of ACE2 against LPS-induced lung injury, the specific MAPKs inhibitors were pretreated intraperitoneally in rats receiving LPS injection. Pretreatment with p38, ERK1/2 or JNK inhibitors significantly attenuated LPS-induced inflammatory response and injury in rat lung with ACE2 knockdown. Moreover, the Mas receptor antagonist or ACE2 inhibitor abolished the protective effects of ACE2 overexpression in LPS-induced protein and cytokine secretion in BALF, which was obviously reversed by specific MAPKs inhibitor. Whereas, only the ERK1/2 inhibitor significantly attenuated lung injury in ACE2 overexpressing rats pretreated with A779. These results suggest that there may be other signaling pathways that are involved in the mechanism of ACE2 protective effects from LPS-induced lung injury.

The NF-κB family, including p65 (RelA), p50/p105 (NF-κB1), p52/p100 (NF-κB2), RelB and c-Rel, exists as homo- or heterodimers in cytoplasm and maintains inactive state by binding to IκB protein. Upon stimulation by LPS or pro-inflammatory cytokines, IκB protein is phosphorylated by activating IκB kinase (IKK) and degraded, leading to activation and then translocation of NF-κB into the nucleus to upregulate transcription of inflammatory genes^47^,^48^. The activation of NF-κB is pivotal to the development of endotoxin-induced ARDS in animals^49^,^50^. Imai *et al*. identified the TLR4-NF-κB signaling pathway as a key role in controlling severity of acute lung injury^24^. In the present *in vivo* study, overexpressing ACE2 in the rat lung significantly suppressed LPS-induced NF-κB p50/p65 phosphorylation and enhanced IκBα expression, which was completely reversed by Mas receptor antagonist or ACE2 inhibitor pretreatment. In contrast with ACE2 overexpression, silencing ACE2 markedly enhanced LPS-induced activation of NF-κB p50/p65 and degradation of IκBα. These results indicate that NF-κB signaling pathway is also important and may cooperate with MAPKs pathway to facilitate the protective effect of ACE2 against LPS-induced ARDS. The result of our *in vitro* study also suggests that ACE2 prevents PMVECs from LPS-induced apoptosis and inflammation by inhibiting JNK and NF-κB activity^25^. Our findings are consistent with previous observations that pharmacological inhibition of ERK or p38 attenuates LPS-induced lung injury *via* suppressing the activation of the NF-kB pathway^18^,^19^.

**Figure 10:**
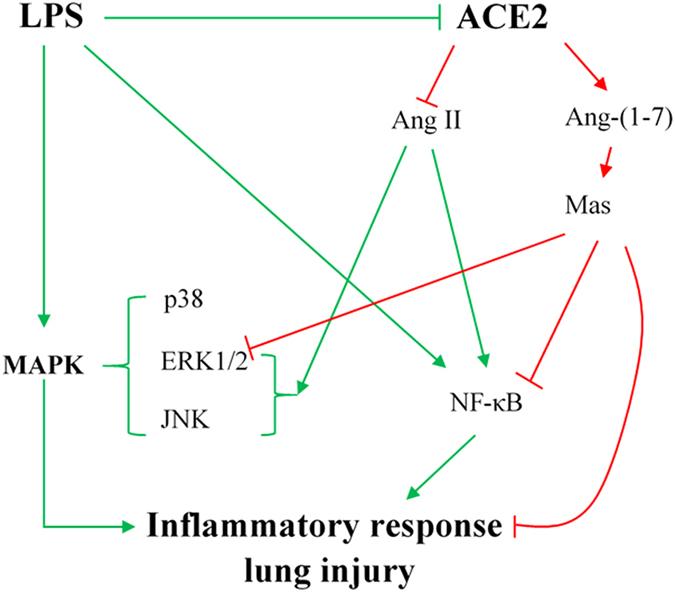
Schematic diagram of the role of ACE2 in LPS-induced acute lung injury in rat.

In summary, the previous studies and the data presented here suggest that AEC2 protects lung injury likely *via* generating Ang-(1-7), which in turn stimulates Mas-mediated signaling to inhibit ERK1/2 and NF-κB activation during the process of ARDS (Fig. 10). Clinically, intravenous administration of recombinant human ACE2 in healthy humans results in increased, decreased or unchanged Ang-(1-7) serum concentration without cardiovascular effects^51^. In a clinical trial performed by Petty WJ *et al*., Ang-(1-7) administration led to a decrease of plasma placental growth factor level in cancer patient with clinical benefit^52^, suggesting that the Ang-(1-7)/Mas pathway is a promising therapeutic target of ARDS.

## Additional Information

**How to cite this article**: Li, Y. *et al*. Angiotensin-converting enzyme 2 prevents lipopolysaccharide-induced rat acute lung injury *via* suppressing the ERK1/2 and NF-κB signaling pathways. *Sci. Rep*. **6**, 27911; doi: 10.1038/srep27911 (2016).
