# Author Correction: Angiotensin-converting enzyme 2 prevents lipopolysaccharide-induced rat acute lung injury via suppressing the ERK1/2 and NF-κB signaling pathways

**DOI:** 10.1038/s41598-021-98272-6

**Published:** 2021-09-14

**Authors:** Yingchuan Li, Zhen Zeng, Yongmei Cao, Yujing Liu, Feng Ping, Mengfan Liang, Ying Xue, Caihua Xi, Ming Zhou, Wei Jiang

**Affiliations:** Department of Anesthesiology, Shanghai Jiaotong University Affiliated Sixth People’s Hospital, Shanghai, 200233 China

Correction to: *Scientific Reports* https://doi.org/10.1038/srep27911, published online 15 June 2016

This Article contains errors.

For Figure 1A, lanes 1–3 were inadvertently duplicated for lanes 10–12. In addition, it was not clearly indicated in the original Figure that this image was a composite. A corrected version of Figure 1 and its figure legend appears below.

**Figure 1:**
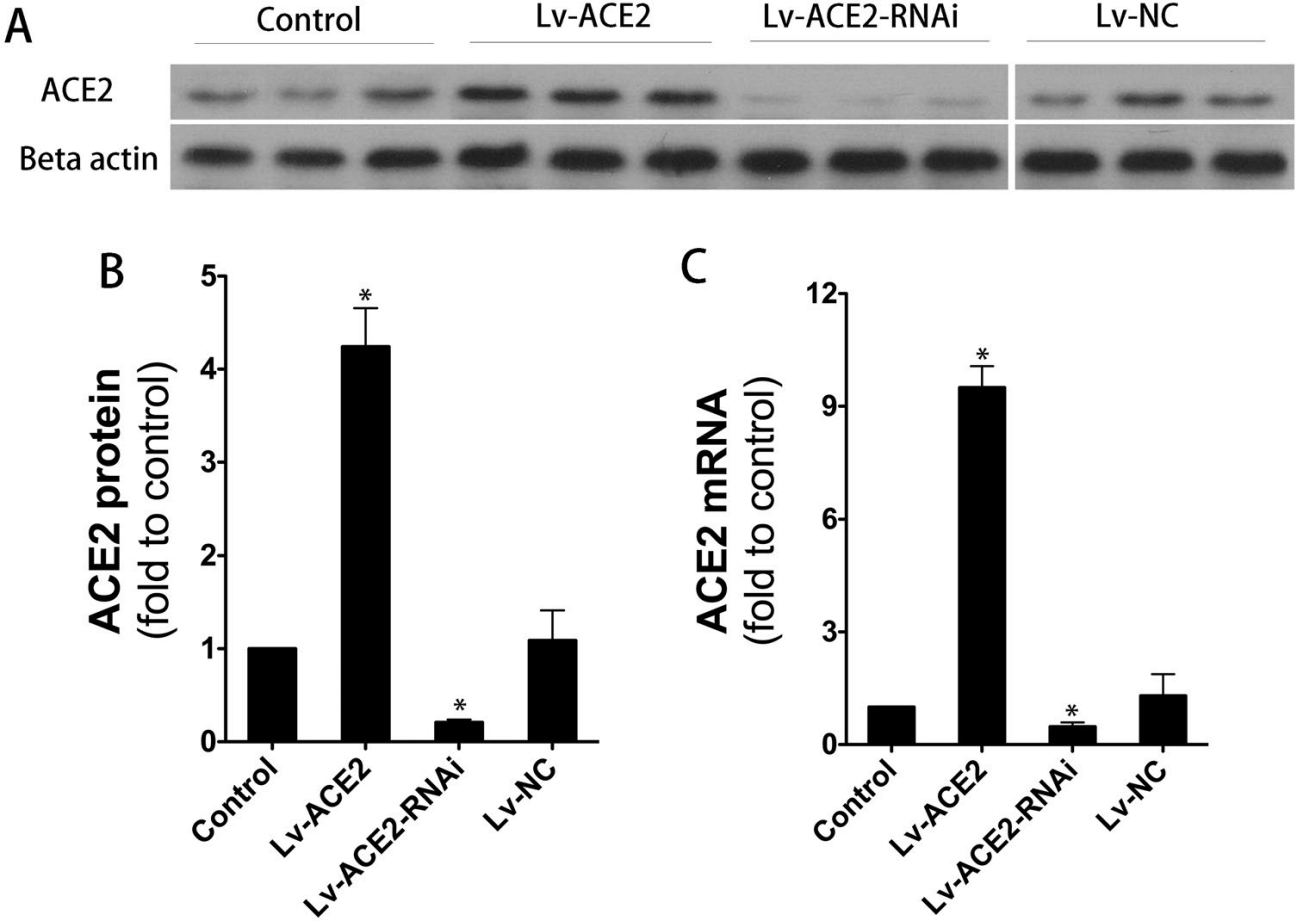
Efficiency of gene transfer at two weeks after Lenti-Ace2 and Lenti-Ace2-RNAi delivery. (**A**,**B**) Western-blotting analysis showed that ACE2 expression of rat lung tissue was significantly increased in the Lv-ACE2 group and decreased in the Lv-ACE2-RNAi group, as compared with the control group. (**C**) Quantitative analysis of ACE2 mRNA levels by using RT-PCR. Lung ACE2 mRNA levels were significantly increased by Lenti-ACE2 transfection, which were suppressed by Lenti-ACE2-RNAi transduction. The Data are represented as mean ± SD. *p < 0.05, versus control group (n = 3, per group). Please note that for **A**, lanes 1–9 and lanes 10–12 are from two gels run in parallel. A single loading control was used, for the gel carrying lanes 1–9.

