# Author Correction: Angiotensin-converting enzyme 2 prevents lipopolysaccharide-induced rat acute lung injury via suppressing the ERK1/2 and NF-κB signaling pathways

**DOI:** 10.1038/s41598-022-09404-5

**Published:** 2022-03-29

**Authors:** Yingchuan Li, Zhen Zeng, Yongmei Cao, Yujing Liu, Feng Ping, Mengfan Liang, Ying Xue, Caihua Xi, Ming Zhou, Wei Jiang

**Affiliations:** Department of Anesthesiology, Shanghai Jiaotong University Affiliated Sixth People’s Hospital, Shanghai, 200233 China

Correction to: *Scientific Reports* https://doi.org/10.1038/srep27911, published online 15 June 2016

This Article contains errors.

For Fig. 6C, the lanes shown for JNK are incorrect. The correct Fig. 6 and accompanying legend appear below.

**Figure 6:**
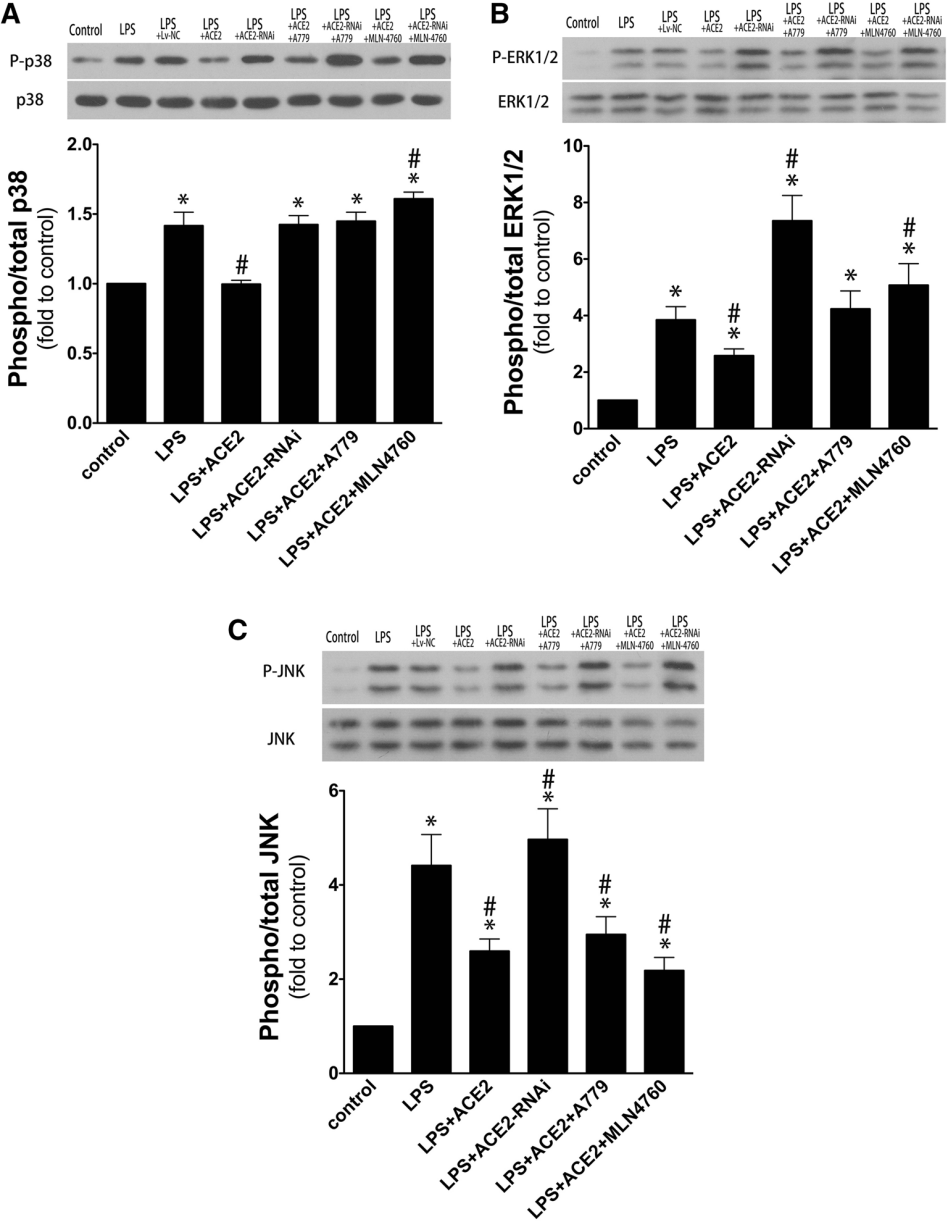
Effects of ACE2 and different treatments on the phosphorylation of MAPKs in lung tissue. LPS exposure caused a marked increase of the phosphorylation levels of p38 MAPK (**A**), ERK1/2 (**B**) and JNK (**C**), which was significantly suppressed by ACE2 overexpression in rat lung. Pretreatment with A779 or MLN-4760 completely abolished the inhibitory effects of ACE2 overexpression on LPS-induced p38 MAPK and ERK1/2 phosphorylation, but did not affect the level of JNK phosphorylation. ACE2 RNAi in rat lung significantly enhanced the LPS-induced ERK1/2 and JNK phosphorylation but did not change p38 MAPK phosphorylation. Data are represented as mean ± SD. *p < 0.05, versus control group; ^#^*p* < 0.05, versus LPS group; ^$^*p* < 0.05, versus ACE2 group (n = 6, per group).

In addition, full blots for the data presented were omitted in the original Article. They have been included in the attached Supplementary Information file.

## Supplementary Information

Supplementary Information.

