# Supplementary material for: Author Correction: Angiotensin-converting enzyme 2 prevents lipopolysaccharide-induced rat acute lung injury via suppressing the ERK1/2 and NF-κB signaling pathways
Source: Sci Rep. 2022 Mar 29;12:5310. doi: 10.1038/s41598-022-09404-5 (PMC8964692; doi:10.1038/s41598-022-09404-5)
Supplement: Supplementary file 1 — Supplementary Information. [file 41598_2022_9404_MOESM1_ESM.docx]

Angiotensin-converting enzyme 2 prevents lipopolysaccharide induced rat acute lung injury *via* suppressing the ERK1/2 and NF-κB signaling pathways

Yingchuan Li, Zhen Zeng, Yongmei Cao, Yujing Liu, Feng Ping, Mengfan Liang, Ying Xue,

Caihua Xi, Ming Zhou & Wei Jiang

Department of Anesthesiology, Shanghai Jiaotong University Affiliated Sixth People’s Hospital, Shanghai 200233, China.

Correspondence to W.J. (email: jiangw@sjtu.edu.cn)


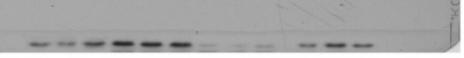
ACE2


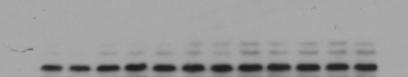
β-actin

**Figure S1**. The originals of western blots for Figure 1.


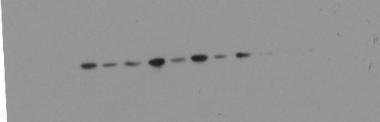
 ACE2


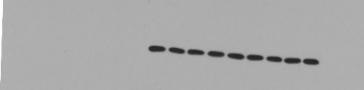
 β-actin

**Figure S2**. The originals of western blots for Figure 4


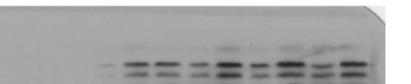
P-ERK


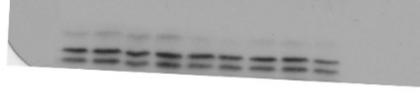
 ERK


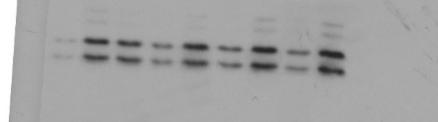
P-JNK


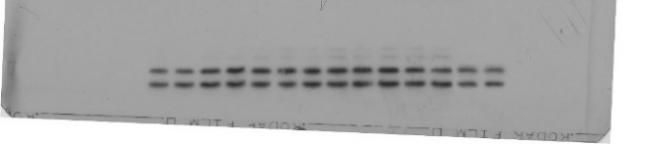
JNK


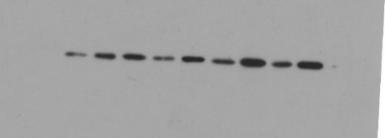
P-p38


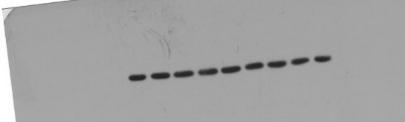
p38

**Figure S3**. The originals of western blots for Figure 6.


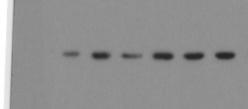
P-p65


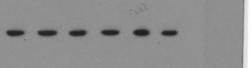
 p65


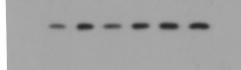
 P-p50


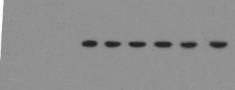
 p50


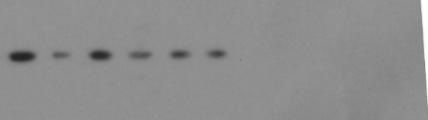
IκBα


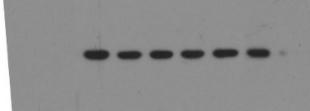
β-actin

**Figure S4**. The originals of western blots for Figure 9.
